# A hSCARB2-transgenic mouse model for Coxsackievirus A16 pathogenesis

**DOI:** 10.1186/s12985-021-01557-5

**Published:** 2021-04-21

**Authors:** Yanli Chen, Heng Li, Jinxi Yang, Huiwen Zheng, Lei Guo, Weiyu Li, Zening Yang, Jie Song, Longding Liu

**Affiliations:** 1 Institute of Medical Biology, Yunnan Key Laboratory of Vaccine Research and Development On Severe Infectious Diseases, Chinese Academy of Medical Science and Peking Union Medical College, No. 935 Alternating Current Road, Wuhua District, Kunming, 650118 Yunna China; 2 Key Laboratory of Systemic Innovative Research on Virus Vaccine, Chinese Academy of Medical Sciences, Kunming, 650118 China

**Keywords:** Coxsackievirus A16 (CA16), Hand, Foot and mouth disease (HFMD), Human scavenger receptor class B, member 2 (hSCARB2), Respiratory and neurological pathology

## Abstract

**Background:**

Coxsackievirus A16 (CA16) is one of the neurotropic pathogen that has been associated with severe neurological forms of hand, foot, and mouth disease (HFMD), but its pathogenesis is not yet clear. The limited host range of CA16 make the establishment of a suitable animal model that can recapitulate the neurological pathology observed in human HFMD more difficult. Because the human scavenger receptor class B, member 2 (hSCARB2) is a cellular receptor for CA16, we used transgenic mice bearing human SCARB2 and nasally infected them with CA16 to study the pathogenicity of the virus.

**Methods:**

Coxsackievirus A16 was administered by intranasal instillation to groups of hSCARB2 transgenic mice and clinical signs were observed. Sampled at different time-points to document and characterize the mode of viral dissemination, pathological change and immune response of CA16 infection.

**Results:**

Weight loss and virus replication in lung and brain were observed in hSCARB2 mice infected with CA16, indicating that these animals could model the neural infection process. Viral antigens were observed in the alveolar epithelia and brainstem cells. The typical histopathology was interstitial pneumonia with infiltration of significant lymphocytes into the alveolar interstitial in lung and diffuse punctate hemorrhages in the capillaries of the brainstem. In addition, we detected the expression levels of inflammatory cytokines and detected high levels of interleukin IL-1β, IL-6, IL-18, and IFN-γ in nasal mucosa, lungs and brain tissues.

**Conclusions:**

The hSCARB2-transgenic mice can be productively infected with CA16 via respiratory route and exhibited a clear tropism to lung and brain tissues, which can serve as a model to investigate the pathogenesis of CA16 associated respiratory and neurological disease.

## Introduction

Coxsackievirus A16 (CA16) is a member of the Human enterovirus A (HEV-A) species of the Enterovirus genus of picornaviridae, and it is one of the major pathogens associated with hand, foot, and mouth disease (HFMD) in infants and young children besides Enterovirus A71 (EV71) [1, 2]. HFMD caused by CA16 infection is generally thought to cause mild and self-limiting symptoms, such as blisters/ulcers on the hands and feet and in the mouth as well as pharyngitis in infants and children. However, increasing evidences show poor clinical outcomes in patients infected with CA16 [3–7], such as fatal myocarditis, pneumonia, aseptic meningitis and encephalitis, which make clinical treatment and prevention challenging. The precise mechanisms of CA16-mediated disease, particularly the pathogenesis of central nervous system (CNS), have not yet been fully understood because suitable and relevant animal models have not been established.

In humans, the main route of CA16 infection is through the oral (OL) route, but the respiratory route has also been documented and became an important natural route of infection [8–11]. Most of the previous animal models, including murine, adult mice and gerbil models were inoculated with this virus via an intraperitoneal (i.p.) [12–14] or intracerebral (i.c.) [15] route. These animals mainly demonstrated an infection process occurred in skeletal and cardiac muscle tissues and replication profile with obvious signs of hind-limb paralysis. Nevertheless, since these inoculation routes were not the natural route for CA16 infection and no neurological lesions were observed, the application of these models is limited. Several studies tried to establish animal models that can reproduce human neurological pathogenesis via natural infective route including oral and respiratory route. In recent studies, 21-day-old gerbils [16] and 7-day-old hamsters [17] were used to establish the orally infected animal models. However, gerbils exhibited lower infection efficiency in detected tissues and no obvious disease symptoms were observed in the CNS, which appeared to be rather resistant to CA16 infection. Hamsters could develop neurological disease by inoculating of the mouse-adapted strains, but it should be noted that mouse-adapted strains are unable to represent all the typical characters of clinical viruses. As for the respiratory infection animal models, our group has developed large animal models including tree shrew [18] and rhesus macaques [19] to study the pathological mechanisms of neurological lesions, but their use are limited for ethical and economic reasons and few studies have focused on respiratory route with respect to small animal thus far. Therefore, we would like to further investigate the suitability of small animals to study CA16 infections via respiratory route based on our previous work.

It is generally believed that specific cellular receptors determine the host range specificity and tissue tropism for most animal viruses. Similar to poliovirus and EV71, CA16 has a limited host range and humans are the only known natural host [20, 21]. Human scavenger receptor class B, member 2 (hSCARB2) has been demonstrated to be a candidate cellular receptor for CA16 and EV71 [21–25]. SCARB2, also known as lysosomal integral membrane protein-2, localizes mainly to lysosomes and acts as a receptor for lysosomal targeting of β-glucocerebrosidase [26–28]. Previously, successful in vivo EV71 infection and pathogenesis have been achieved by intraperitoneal (i.p.) inoculation of adaptive viral strains into hSCARB2 transgenic mice [29–31]. Both EV71 and CA16 belong to the enterovirus genus and cause similar clinical symptoms. However, no study has demonstrated thus far whether CA16 is able to infect transgenic mice expressing hSCARB2 as well. Here, we assess the utility of the transgenic mouse as a model for investigating the mode of CA16 dissemination, tissue tropism and pathology within the host via the respiratory infection route. The data obtained further extend our knowledge of CA16 infectious disease pathology in general and the CNS pathology in particular.

## Material and methods

### Ethics statement

Transgenic C57BL/6 J mice expressing hSCARB2 were purchased from the National institutes of Food and Drug Control of China. The hSCARB2 transgenic mice were generated in accordance with previously described methods [32]. All mice were housed in a high-efficiency particulate air-filtered individual isolation unit in an Animal Biosafety Level 2-enhanced (ABSL-2 +) facility, which complied with the requirements for mouse housing, environment, and comfort as described in the Guide for Laboratory Animals Care issued by the Institute of Medical Biology. The Yunnan Provincial Experimental Animal Management Association and the institutional Experimental Animal Ethics Committee approved the experimental protocols.

### Mouse study design

A total of 20 transgenic mice (weight: 17.00–20.00 g, 4 weeks old) were randomly divided into the mock control and CA16 groups. Another 15 C57BL/6 J mice (weight 17.00–20.00 g, 4 weeks old) from the Animal Center of Chinese Academy of Medical Sciences served as infected control to compare the susceptibility to CA16 infection with hSCARB2 transgenic mice. Based our earlier work on nasally infected CA16 tree shrew [18] and rhesus macaques model [19], fifteen transgenic mice and fifteen C57BL/6 J mice were infected with 20 μl of CA16-GX/20 strain (2 × 10^4.5^ CCID_50_) via the nostrils dropwise. The CA16 virus strain (sub-genotype B) was isolated from a throat swab from an HFMD patient obtained in Guangxi in 2010 (GenBank: JN590244.1) and grown in Vero cells (ATCC, Manassas, VA, USA), which were maintained in Dulbecco's Modified Eagle Medium (DMEM, HyClone, Logan, UT, USA) supplemented with 10% fetal bovine serum (FBS, Gibco, Grand Island, NY, USA). While 5 hSCARB2 transgenic mice used as mock controls were inoculated with the same dosage of phosphate-buffered saline (PBS) via the same route.

After inoculation, the animals were monitored daily for survival and clinical manifestation for 21 days. The onset and duration of all visible changes, such as reduced mobility, limb weakness, paralysis and death, were recorded. Animal feces, and throat swabs were collected daily to detect viral load. Three hSCARB2 transgenic mice and three WT mice were respectively sacrificed on days 3, 7, 12, 15 and 21 after infection, and the organs or tissues were harvested for viral distribution analysis, histopathology, immunohistochemistry and inflammatory cytokines detection. For nasal washes, a 22-gauge catheter was inserted into the posterior naris from the opening of the trachea and along the direction of the nostrils. 1 ml of pre-warmed sterile saline solution was perfused gently into the nasal cavities, lavage fluid was collected from the anterior naris, centrifuged at 220 × g and 4 °C for 10 min, and the supernatant was stored at − 20 °C. For throat swabs, holding a sterile plain swab in the mouse's mouth and pushing the swab into the throat for a few seconds. The swab will collect a sample of the secretions being produced in the back of the mice’s throat and then soaked in sterile-filtered PBS. For nasal mucosa, facial disinfection was implemented and nasal mucosa was obtained under sterile environment by resecting of mucosa from nasal septum exposed by extending incision of nasal skin and bone below nares to inner cavity. Nasal mucosa was carefully removed from the nasal septum mucosa and perfused into PBS. Then mouse’s hind legs and small intestine were collected for experiment.

### Real-time PCR test for viral load quantity

For viral load examination, total RNA was extracted from equivalent weights of tissue samples and volumes of blood samples from infected and control mice with TRIzol reagent (TianGen Biotech, Co., Ltd., Beijing, China) according to the manufacturer’s instructions. The total RNA was eluted in a final volume of 30 µL. Then, we established a standard curve for cycle thresholds (Cts) versus virus copy number by measuring the serially diluted concentrations of the CA16 RNA standards generated from the in vitro transcription of a DNA gene fragment containing the CA16 vp1 gene region. For quantification, real-time TaqMan RT-PCR assay was performed using the TaqMan one-step RT-PCR Master Mix in the CFX96 Touch™ Real-Time PCR Detection system (Bio-Rad, Laboratories, Hercules, CA, USA). The experiments were carried out by adding the primer (200 nm), FAM/TAMRA probe (100 nm) (TAKARA Biotechnology Co., Ltd., Dalian, China), and 2 µL of RNA into the TaqMan PCR mater mix, for which the total reaction volume is 20µL. The following sequences including CA16-specific primers and probe: forward primer, 5′-CTAGTAGTCACAGATTAGGCACTGGTG-3′; reverse primer 5′-CATTGTGATGATGCTGACAAGACC-3′ and the probe 5′FAM-CGTCTAATGCTAGCGACAA-TAMRA-3′; The following reaction conditions were applied for all PCR experiments: 5 min at 42 °C and 10 s at 95 °C, followed by 40 cycles at 95 °C for 5 s, and 60 °C for 30 s. The copy number for each sample was calculated based on the standard curve and Ct values of the samples. Equivalent volumes of RNA copies and equivalent volumes of tissue weights (or equivalent volumes of blood) thus can be converted to RNA copies/mg tissue or ml blood.

### Histopathological and immunohistochemical (IHC) staining

Tissue samples from sacrificed mice were fixed in 10% formaldehyde, dehydrated, embedded, and then cut into 4-μm-thick sections for hematoxylin and eosin (HE) staining assays. For immunohistochemical analysis, the sections were prepared according to the manufacturer’s protocol. Briefly, the slides were deparaffinized, hydrated, antigen-repaired, and then blocked in 4% BSA. CA16 antigen was detected using an anti-enterovirus 71 antibody and cross-reacted with CA 16 antibody (Cat # MAB979, Millipore) prepared by diluting 1:1000 in PBS containing 1% BSA. These slides were washed with PBST and incubated with goat poly-HRP anti-rabbit IgG antibody (Cat # AS040, AB clonal) as a secondary antibody for 35 min at 37 °C. Peroxidase activity was detected with an Enhanced HRP-DAB Chromogenic Substrate Kit (TianGen Biotech, Co., Ltd., Beijing, China). Finally, the slides were examined under a light microscope.

### Quantification of cytokine mRNA

RNA isolations were performed on mouse lung tissue samples with the TRNzol-A + Reagent kit (TianGen Biotech, Co., Ltd., Beijing, China) according to the manufacturer’s protocols. Then, cytokine expression levels were normalized to Beta-actin (β-actin) and are reported as the fold change compared with mock-infected animals. Primer sequences for IL-1β [29], IL-6 [31], IL-18 [33], TNF-γ [34]and β-actin [29] were published elsewhere. Quantitative real-time PCR (qRT-PCR) was performed by using a CFX96 Touch™ Real-Time PCR Detection system (Bio-Rad, Laboratories), and a One Step SYBR PrimeScript™ RT-PCR Kit (TAKARA Biotechnology Co., Ltd.). Each reaction consisted of 1 cycle of 42 °C for 5 min, 95 °C for 10 s, followed by 40 cycles of 95 °C for 5 s and 60 °C for 30 s. The results of cytokines expression were normalized by β-actin, respectively, and calculated using the 2^−ΔΔCT^ method [35].

### Neutralization antibody titer test

To investigate the dynamic changes of neutralizing antibody response to CA16 in hSCARB2 transgenic and WT mice after infection, serum samples were collected at 3, 7, 12, 14, and 21 days post-infection. CA16-neutralizing antibodies were analyzed using a standard protocol. Briefly, mouse serum was heat-inactivated for 30 min at 56 °C, then diluted 1:2 in minimum essential medium containing 2% fetal bovine serum (FBS) (Gibco, Life Technologies, Shanghai, China). After that, diluted serum was transferred in triplicate to the first row of one 96-well plate and then diluted two-fold from 1:2 to 1:512. 100 CCID_50_ were combined with the diluted sera in a 96-well plate and incubated at 37 °C for 3 h before adding 10,000 Vero cells/well. After incubation, the mixtures were added onto a monolayer of Vero cells and the cells were inspected daily for cytopathic effect (CPE) for up to 4 days. Neutralizing antibody titers were taken to be the highest dilution of serum that inhibited 50% of the viral growth. Neutralization titers were estimated with the Spearman–Karber method and expressed in log2 form (e.g., 4 is a titer of 1:16).

### Statistics

GraphPad Prism 8 (Version 8.0, La Jolla, CA, USA) was used to graph data and to perform statistical analyses. Kaplan–Mei survival curves were compared by the log-rank test. To compare cytokine expression levels between groups, the Mann–Whitney U test was used. Means ± SEMs (standard errors of the mean) were graphed and *p* < 0.05 was considered to be statistically significant.

## Results

### Clinical observations of CA16-infected hSCARB2-transgenic mice

**Fig. 1:**
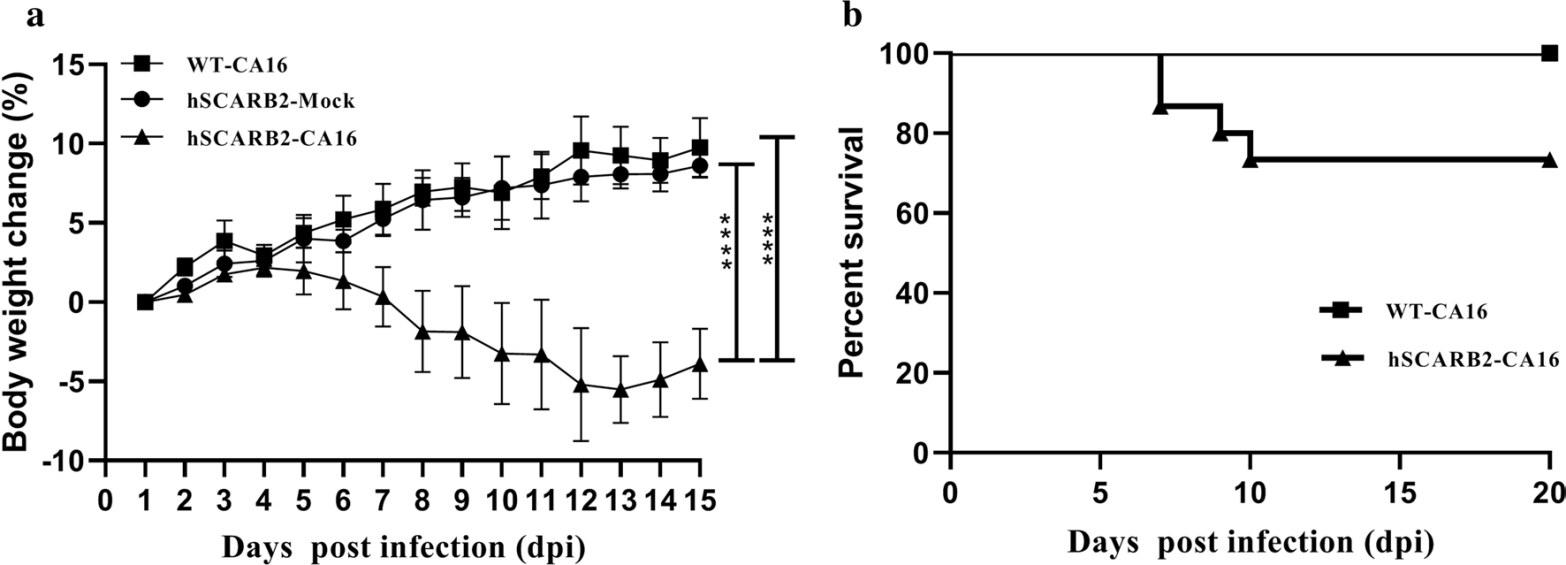
Weight loss and survival rates in infected and mock control mice. **a** Weight loss was recorded for 15 days. hSCARB2 transgenic mice (n = 15) and WT mice (n = 15) were experimentally nasally infected with CA16, and WT and the hSCARB2-Mock mice (n = 5) were used as a control. According to two-tailed t test, weight loss in hSCARB2 mice displayed a significant decline compared with that in hSCARB2-Mock mice or WT mice (****p < 0.0001). **b** Survival curves for hSCARB2-Mock mice and WT mice

Compared to mock-infected mice and WT mice, slight bristled fur and weight loss were observed in hSCARB2-transgenic mice during the 15 days of observation, and other clinical symptoms such as limb weakness and paralysis were not found. No weight loss was observed in infected WT mice, similar to uninfected hSCARB2-transgenic mice which progressively gained weight over the course of the experiment. However, infected hSCARB2-transgenic mice lost weight progressively from 4 to 13 days post-infection reaching 5% weight loss (Fig. 1a). Generally, patients infected with this virus can appear to have various disease severities ranging from mild symptoms, such as cold-like clinical signs and blisters/ulcers in the oral mucosa and limbs, to severe respiratory and neurological infections, including aseptic meningitis, encephalitis and even fatal myocarditis and pneumonia [36, 37]. In our study, however, the most obvious clinical signs was weight loss and 4 of 15 infected animals died during the observation. In contrast, WT mice exhibited 100% survival rate (Fig. 1b). And hSCARB2-Mock mice appeared to be healthy throughout the course of the experiment.

### Dynamic profile of CA16 viral load in tissues of nasally infected hSCARB2-transgenic mice

**Fig. 2:**
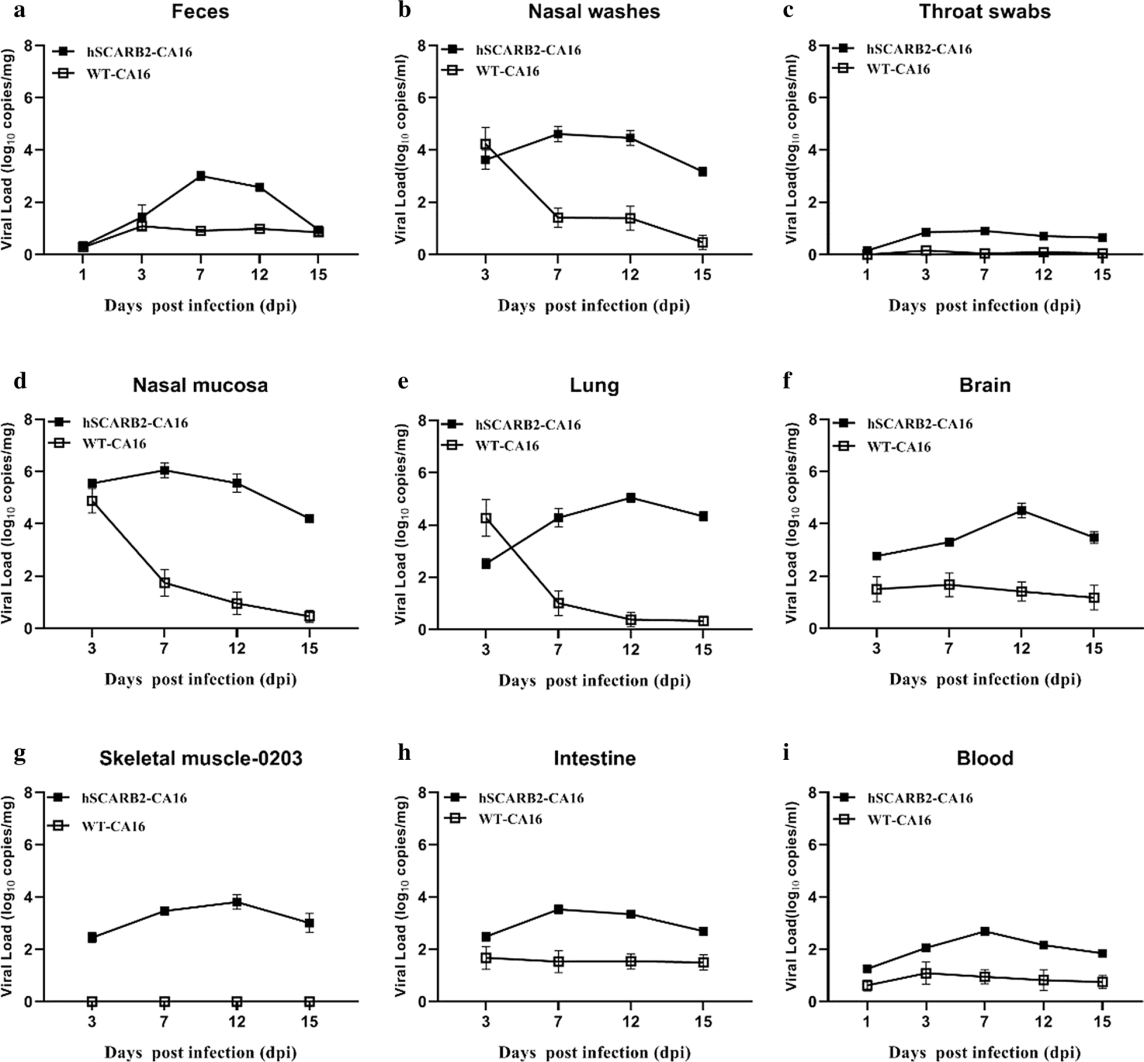
Dynamic distribution of CA16 in infected hSCARB2-transgenic and WT mice through the respiratory route. Viral loads in feces (**a**) and nasal washes (**b**), throat swabs (**c**), nasal mucosa (**d**), lung (**e**), brainstem (**f**), skeletal muscle (**h**), intestine (**i**) and blood (**j**) of infected hSCARB2-transgenic mice were detected at 3, 7, 12 and 15 dpi. Results are expressed as viral RNA copies/mg tissue or ml blood. Data represent the means ± SEM of results of three mice

To determine the mode of viral dissemination in vivo post respiratory-route infection of WT and hSCARB2-transgenic mice, the viral loads in the feces, nasal mucosa, throat swabs, lungs, blood, intestines and limb muscles of CA16-infected mice at 3, 7, 12 and 15 dpi were detected by real-time PCR. As shown in Fig. 2, virus replication in hSCARB2-transgenic mice was observed in almost all of the tissues assayed except for throat swabs. At an early stage of infection (3 dpi), the highest viral load was detected in nasal mucosa, which is the site of viral infection, with levels of 354,813 copies per mg sample. With the progress of the infection, virus from the sites of infection spread to and replicated in other tissues, including lungs, blood, brainstem, intestines, skeletal muscles and feces, then reduced gradually after reaching a peak value at 7 or 12 days post-infection. Notably, the viral load in the lung (10^5.04^ copies/mg) and brain (10^4.51^ copies/mg) was 1.78 and 1.25 logs higher than that in the muscles (10^3.26^ copies/mg) (Fig. 2e, f, h), despite all of them peaked on the 12 days post-infection in hSCARB2-transgenic mice, which is quite different from the mode of viral dissemination in previous studies evaluating intra-peritoneally or intra-cerebrally infected animals [12, 15]. For WT mice, viral loads in nasal washes (10^4.23^ copies/mL), nasal mucosa (10^4.88^ copies/mg) and lung tissue (10^4.27^ copies/mg) at 3 dpi were the highest of all tissues tested and all decreased to less than 10^2^ copies/mg at 7dpi. Relatively low viral loads (0.0–1.0 × 10^2^ copies/mg or ml) were found in brainstem, intestine, blood and feces. While viral loads in the throat swabs and muscles were barely detectable at all time points tested. These findings demonstrated that hSCARB2-transgenic mice were more susceptible to CA16 infection than WT mice and the virus exhibited a clear tropism for lung and brain tissues in transgenic mice.

### Tissue distribution of VP1 of CA16 in hSCARB2-transgenic mice

**Fig. 3:**
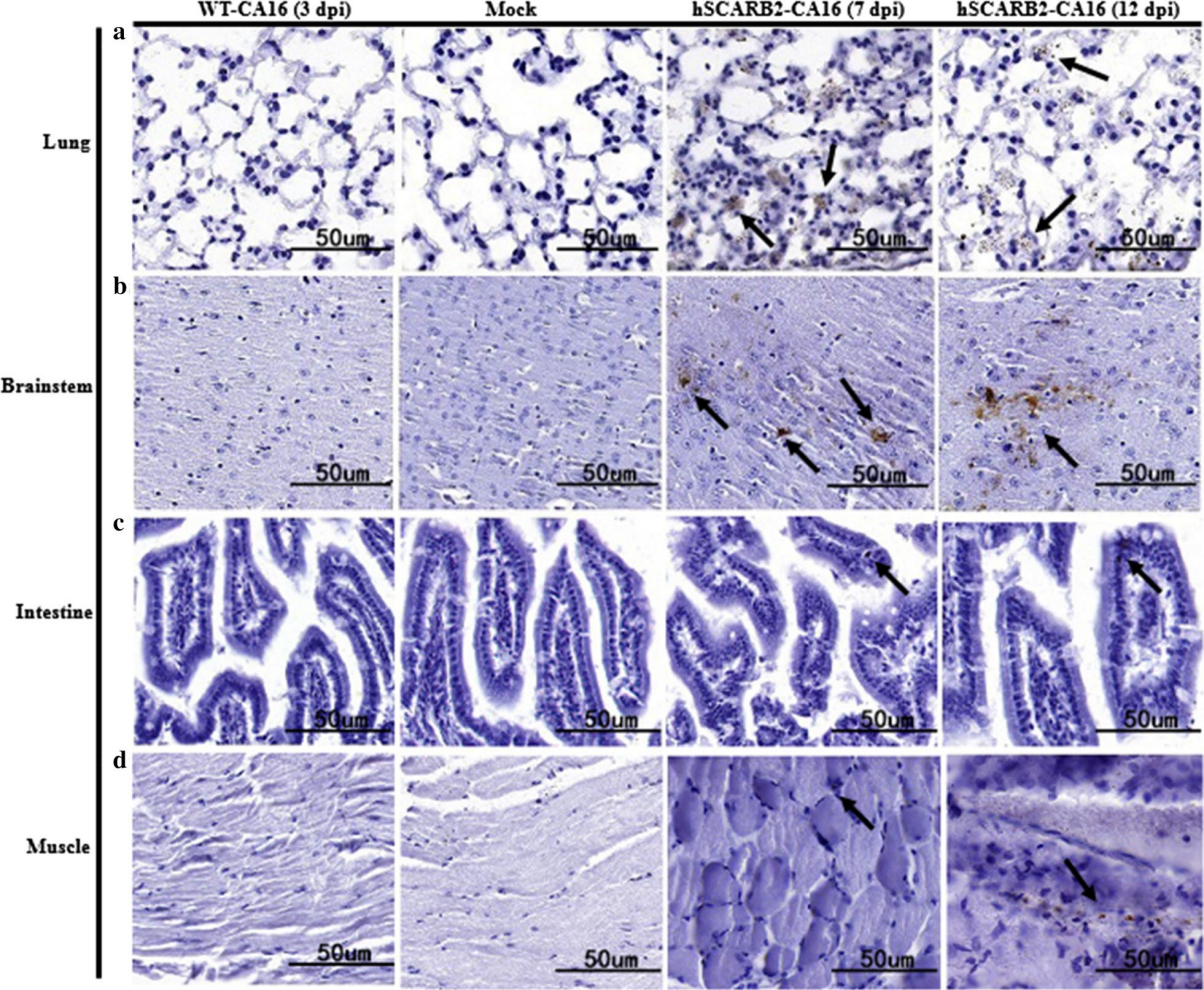
Immunohistochemical results for infected and mock control mice. Representative sections are shown. Infected mice exhibited viral antigen-positive areas (black arrow) in lung (**a**), brainstem (**b**), intestine (**c**) and muscle (**d**). In contrast, no viral antigen was observed in the mock control and WT mice. Observations were made at a magnification of 40; Bar 50 μm

To understand the distribution of virus antigen, the brainstem, lung, skeletal muscle and intestinal tissues of CA16 infected mice were harvested for immunohistochemistry (IHC) examinations. As shown in Fig. 3a, virus antigen was detected in lung and brain at 7 dpi (Fig. 3b) in hSCARB2-transgenic mice. However, limited CA16 antigen was observed in the skeletal muscle and intestinal tissues in hSCARB2-transgenic mice (Fig. 3c, d), and negative reactions were observed in the WT mice during observation. This finding further confirmed that nasally infected hSCARB2 transgenic mice had a tropism to lung and brain tissues rather than to muscle tissues in nasally infected hSCARB2 transgenic mice.

### Pathological changes in hSCARB2-transgenic mice

**Fig. 4:**
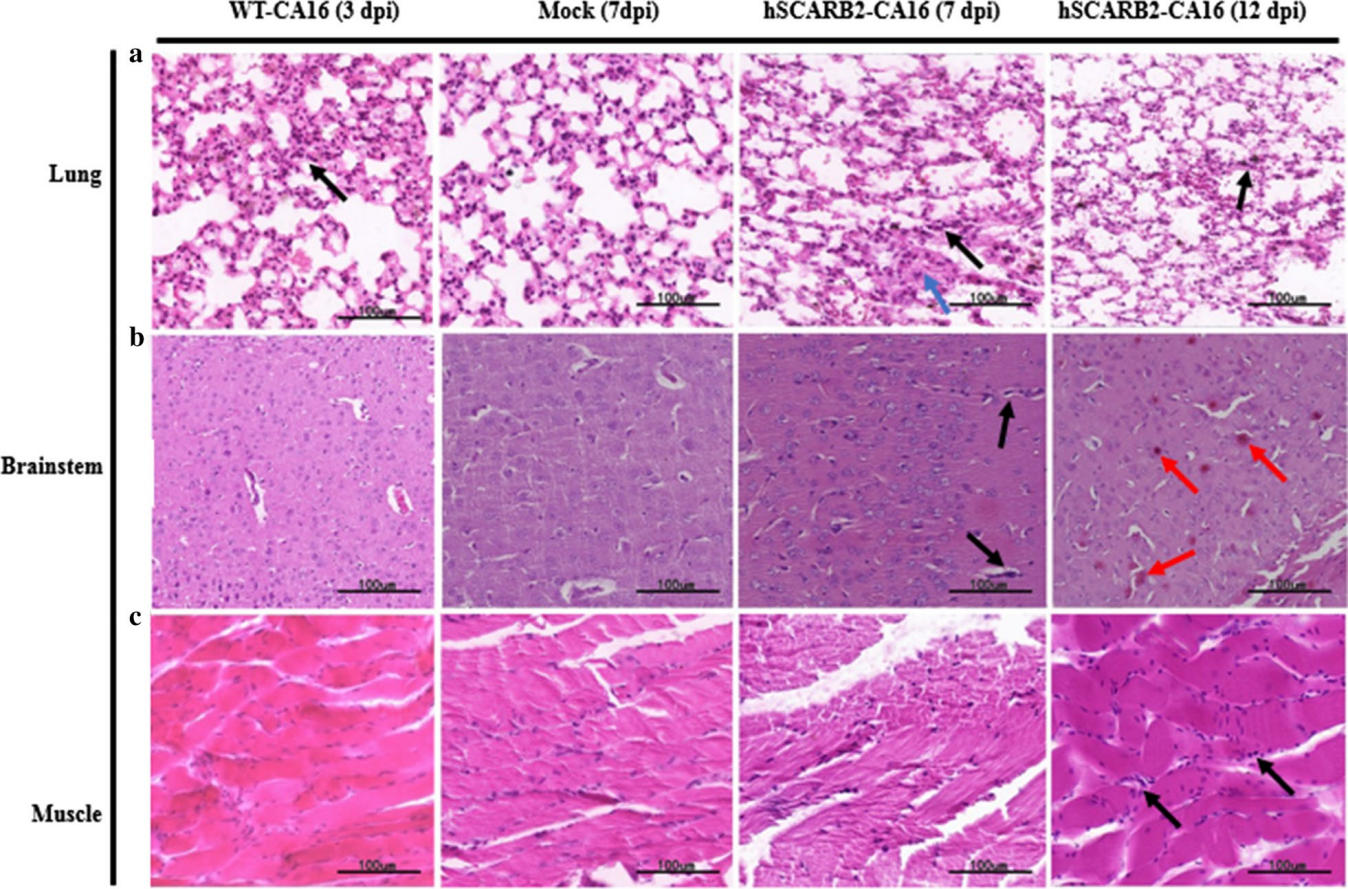
Histologic examination results for infected and mock control mice. The clinical pathological features of CA16 infection in mouse lung (**a**), brainstem (**b**) and muscle tissues (**c**). Inflammatory cell infiltration were shown with black arrows; thickened alveolar septa were shown with blue arrows; punctate haemorrhages were shown with red arrows. Observations were made at a magnification of 40; Bar 100 μm

To investigate the pathological effects of CA16 on nasally infected mice, histopathological examination of the infected hSCARB2 transgenic mice at 7 and 12 dpi and WT mice at 3dpi were carried out. Histologic examination of the lung tissues in hSCARB2 transgenic mice revealed interstitial pneumonia with infiltration of significant lymphocytes into the alveolar interstitium from 7 dpi (Fig. 4a). Microscopically, the lung tissues from transgenic mice displayed interstitial pneumonia characterized by thickened alveolar septa accompanied with infiltration of inflammatory cells in some areas of the lung tissues and accumulation of inflammatory cells in partial alveolar cavities. The brainstem was the most severely affected organs of the central nervous system in the infected mice. At 7dpi, only a few dark blue inflammatory cells are scattered within the brainstem; as the infection progress into the late stage (12 dpi), diffuse punctate hemorrhages in the capillaries were detected in the area of damage (Fig. 4b). However, minor damage was observed for the limb muscles, characterized by a small amount of inflammatory cell infiltration in interstitial cells at 12 dpi (Fig. 4c). However, in WT mice, only weak inflammatory cells infiltration was observed in the pulmonary interstitium at 3 dpi.

### Immune response of CA16 infection in hSCARB2-transgenic mice

**Fig. 5:**
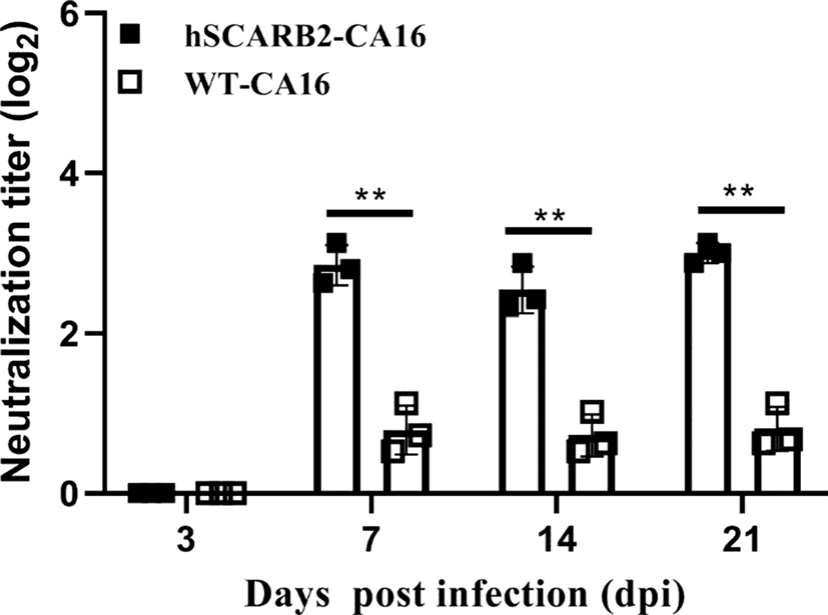
Neutralizing antibody levels in infected mice. Neutralizing antibodies against CA16 were tittered on Vero cells with 100 CCID_50_ virus

**Fig. 6:**
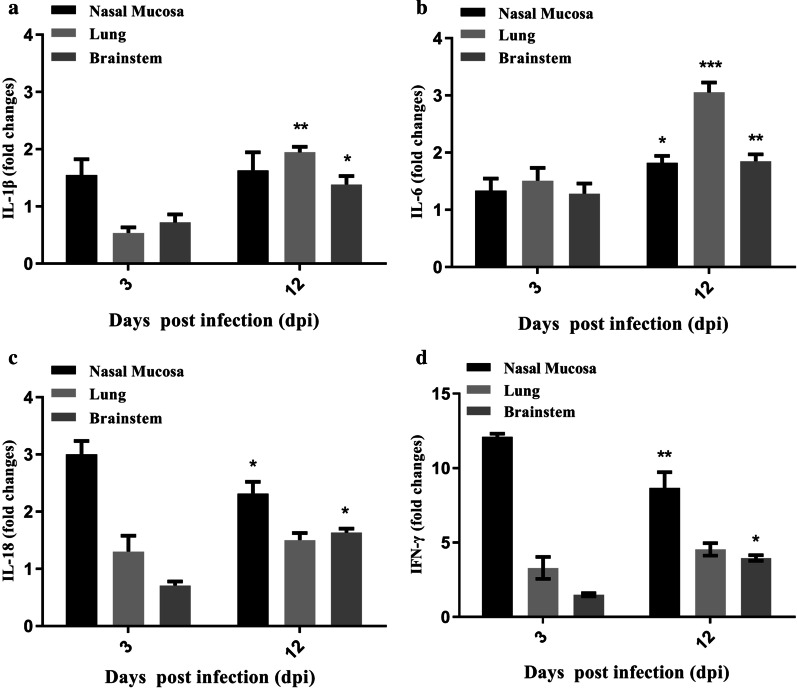
Inflammatory cytokines detection in hSCARB2-transgenic mice after CA16 infection. Cytokines in the nasal mucosa (black), lung (light brown), and brain tissues (dark brown) of infected hSCARB2-transgenic mice were detected at 3 and 15 dpi. Cytokine expression levels were normalized to β-actin and are reported as the fold change compared with uninfected mice. The data are expressed as the means standard errors of the means ± SEMs of three mice. *p < 0.05; **p < 0.01 (relative to the 3dpi group)

To detect the ability of viral infection to elicit an antibody response, three mice of hSCARB2-transgenic and WT mice were followed for up to 21 days after inoculation. The immunological analysis of the CA16-infected hSCARB2-transgenic showed a typical serum antibody response of viral-induced characteristics. Antibodies in hSCARB2-transgenic mice presented on the 7th day post-infection and increased to a peak level of 1:8 at 21 days post-infection, while significantly lower antibody response was detected in WT mice (Fig. 5). Average neutralizing antibodies of hSCARB2-transgenic mice had geometric mean titers of 1:4, suggesting that CA-16 infection elicited an adaptive immune response in transgenic mice. Furthermore, enhanced cytokine production has been demonstrated to represent immune activation in both HFMD patients and mouse models [38–40]. However, the relationship between the viral-specific immune response and cytokine production of CA16 infection remains ill-defined. Here, we focused on IL-1β, IL-6, IL-18 and IFN-γ, since these cytokines are important inflammatory cytokines expressed in HFMD patients. To follow the time course of virus-host interactions post-inoculation, we compared the levels of cytokines at early and late stages of the disease. mRNA expression levels of inflammatory cytokines in the nasal mucosa, lung and brain tissues of the normal control group and the CA16 group were detected by qPCR. Consistently, the levels of IL-1β, IL-6, IL-18 and IFN-γ in lung and brain tissues increased progressively in conjunction with disease advancement, reaching the highest levels at 12 dpi. Unlike the expression patterns in lung and brain, the patterns of IL-18 and IFN-γ in nasal mucosa were identified as early pro-inflammatory cytokines, as their levels were significantly increased at 3 dpi and declined by 12 dpi (Fig. 6).

## Discussion

Previous studies on CA16 nasally infected animal models primarily focused on large animals, with little emphasis on small animal models. It’s well known that the non-human primate (NHP) model best recapitulates CA16 pathogenesis in humans since NHPs present the nearest anatomy, physiology, and the immune system from humans [41–43]. However, it should be noted that these large animal models are generally constrained by significant individual differences, poor repeatability and small sample size. Compared with large animals, small animal models are more convenient and cost-effective to study the mechanism of viral pathogenesis. However, this animal model had never been evaluated for its suitability to study CA16 infections via respiratory route. Thus, establishing the nasal mouse models for CA16 infection is highly desirable, which would be complementary to large animal studies in some aspects.

In the present study, we showed that the exogenous expression of hSCARB2 in mice was sufficient to confer susceptibility to CA16 infection and subsequent development of neuro-pathogenesis, suggesting that hSCARB2 also functions as a cellular receptor for CA16 infection in vivo. In CA16-infected hSCARB2-transgenic mice, although no typical clinical symptoms such as limb weakness and paralysis were observed, other features such as histopathological changes and inflammatory responses could mimic some manifestations in human patients with HFMD.

The viral load found in various organs and tissues indicated that the virus is able to enter the host through respiratory route and replicate in vivo. Consistently, the virus load in the brain in hSCARB2-transgenic mice increased progressively and peaked at 12 dpi suggesting that CA16 is neurotropic. Immunohistological examination further confirmed the presence of CA16 in the lung and brain tissues. Moreover, histopathological examination demonstrated that CA16 infection resulted in significant neurological damage in the brainstem. In contrast, in the previous intraperitoneally or intracerebrally inoculated animal models, no obviously positive CVA16 antigen or pathological change was found in the CNS, which demonstrated that CA16 had no significant neurotropism in these models [12, 15]. Despite some evidence showing that CA16 infection is associated with damage to muscle tissues, we did not observe the apparent relationship between limb paralysis and the presence of Viral RNA in the skeletal muscle from nasally infected mice, which is different from previous studies [34, 44] and also suggested that infection sensitivity differs slightly between respiratory inoculation and the intraperitoneal- or intracerebral- inoculation route. Consistent with previous studies in orally infected immunodeficiency AG129 mice [45], the viral RNA in the limbs suggests that limb paralysis might be a consequence of virus neuro-invasion rather than direct damage to limb muscle. Furthermore, the distribution of hSCARB2 of each tissue was different in mice, with relatively higher levels in lung, intestine and limb muscles, compared with brain tissue [32]. However, in human tissues, the SCARB2 expression was highest in brain, followed by lung and limb muscles [30]. Therefore, despite the fact that the SCARB2 expression levels in brain and muscles of transgenic mice are contrary to that in humans, CA16 infection exhibited neurotropism rather than muscle tropism in the hSCARB2 mice model.

Previous studies demonstrated that elevated antiviral pro-inflammatory or inflammatory cytokines after viral infection present [30] immune activation and contribute to the immunopathogenesis of EV71 infection in both humans and mice [38–40, 46]. Likewise, the levels of pro-inflammatory cytokines implicated in CA16 infection, namely, IL-1β, IL-6, IL-18, and IFN-γ, were found to be significantly elevated in infected hSCARB2 transgenic mice. Furthermore, the nasal mucosa developed higher levels of IL-18 and IFN-γ than did the lung and brain tissues, likely as a consequence of the stronger mucosal immunity in the nasal mucosa. In addition, since the nasal mucosa is the site of viral infection, increased viral RNA replication at early phase activates the host immune system to clear the input virus, thus resulting in an increase of inflammatory cytokines. Consistent with previous studies in EV71-infected mouse models [47], it was also suggested that type I IFNs represent an essential innate defense mechanism for controlling CA16 in hSCARB2 transgenic mice. Thus, it was not surprising to find that stronger IFN responses in hSCARB2 transgenic mice prevent them from being susceptible to CA16 infection.

The limitation in the current hSCARB2 transgenic mouse model is its lack of oral infection. Although both respiratory and fecal–oral routes are natural routes of CA16 infection, they might present with different pathogenesis profiles in terms of virus susceptibility and tissue tropism. Previously, it was reported that 7-day old hamsters were susceptible to CA16 infection via an oral route, particularly when using a mouse-adapted virus strain [17]. Although this hamster model manifests both HFMD-like lesions and encephalomyelitis, it should be noted that host-adapted virus represents the disadvantage of biasing the natural tropism of the pathogen which may render them less relevant in the clinical context. In another orally infected animal model, no obvious clinical manifestations or pathological lesions were observed with young or older gerbils [14, 16], when infected with a clinical isolate strain. Additionally, our group tried oral route to infect tree shrews and rhesus macaques in previous work [18, 19], and found the nasal insufflation was more effective than oral route (data not shown). Similar results were also found in the EV71 infected animal models including hSCARB2 transgenic mice [30]. The mechanism behind the low efficiency of oral route remains unclear, but one possible explanation is that specific oral bottlenecks in gastrointestinal system may affect the mode of virus dissemination. Since a previous study [48] reported that colonic epithelium serve as a physical barrier to limit the virus trafficking from the oral gut to other body sites, including the CNS, upon oral infection with human poliovirus (HPV), a virus closely related to EV71 and CA16. On the other hand, it should be noted that different promoter in transgenic mice may drive different expression pattern of hSCARB2, which might affect the virus susceptibility and tissue tropism. Therefore, it is worth trying to infect current mouse model via oral route in future research. Meanwhile, it is also of great significance to explore the critical factors involved in viral susceptibility present in different natural infection routes, which will allow understanding of important details in the mode of viral dissemination and infection.

Nevertheless, considering their small size, ease of handling and reduced cost of the mice in comparison to NHPs, this nasally infected hSCARB2-transgenic mouse model also represents an important step toward the development of a suitable animal model of CA16 infection and an improved platform that could facilitate the development of antiviral research in clinical medicine.

## Conclusion

In summary, the results presented in this paper demonstrate that hSCARB2-transgenic mice can be productively infected with CA16 via respiratory route. The virus exhibits a clear neurovirulence, causing neurological lesion-related symptoms in mice, which resembled the symptoms observed in human patients. This hSCARB2-transgenic mice model could further our understanding of neurotropism, neurovirulence, as well as neuropathology of CA16 infection.

## Data Availability

All data presented in this manuscript is included in the text.

## Abbreviations

CA16: Coxsackievirus A16
HFMD: Hand, foot, and mouth disease
hSCARB2: Human scavenger receptor class B, member 2
HEV-A: Human enterovirus A
EV71: Enterovirus A71
CNS: Central nervous system
CCID_50_: 50% Cell culture infectious doses
DMEM: Dulbecco's Modified Eagle Medium
FBS: Fetal bovine serum
PBS: Phosphate-buffered saline
RT-PCR: Real time polymerase chain reaction
HE: Hematoxylin and eosin
CPE: Cytopathic effect
SEM: Standard errors of the mean
dpi: Days post infection
IHC: Immunohistochemistry
NHP: Non-human primate
